# FGF8 morphogen gradients are differentially regulated by heparan sulphotransferases Hs2st and Hs6st1 in the developing brain

**DOI:** 10.1242/bio.028605

**Published:** 2017-11-20

**Authors:** Wai-Kit Chan, David J. Price, Thomas Pratt

**Affiliations:** Centre for Integrative Physiology, Edinburgh Medical School Biomedical Sciences, The University of Edinburgh, Edinburgh, EH8 9XD, UK

**Keywords:** Heparan sulphotransferase, Hs2st, Hs6st1, Erk, Mapk, Neural development, Telencephalon, Fibroblast growth factors, Mouse

## Abstract

Fibroblast growth factor (FGF) morphogen signalling through the evolutionarily ancient extracellular signalling-regulated kinase/mitogen activated protein kinase (ERK/MAPK) pathway recurs in many neural and non-neural developmental contexts, and understanding the mechanisms that regulate FGF/ERK function are correspondingly important. The glycosaminoglycan heparan sulphate (HS) binds to FGFs and exists in an enormous number of differentially sulphated forms produced by the action of HS modifying enzymes, and so has the potential to present an extremely large amount of information in FGF/ERK signalling. Although there have been many studies demonstrating that HS is an important regulator of FGF function, experimental evidence on the role of the different HS modifying enzymes on FGF gradient formation has been lacking until now. We challenged *ex vivo* developing mouse neural tissue, in which HS had either been enzymatically removed by heparanase treatment or lacking either the HS modifying enzymes Hs2st (*Hs2st^−/−^* tissue) or Hs6st1 (*Hs6st1^−/−^* tissue), with exogenous Fgf8 to gain insight on how HS and the function of these two HS modifying enzymes impacts on Fgf8 gradient formation from an exogenously supplied source of Fgf8 protein. We discover that two different HS modifying enzymes, *Hs2st* and *Hs6st1*, indeed differentially modulate the properties of emerging Fgf8 protein concentration gradients and the Erk signalling output in response to Fgf8 in living tissue in *ex vivo* cultures. Both Hs2st and Hs6st1 are required for stable Fgf8 gradients to form as rapidly as they do in wild-type tissue while only Hs6st1 has a significant effect on suppressing the levels of Fgf8 protein in the gradient compared to wild type. Next we show that Hs2st and Hs6st1 act to antagonise and agonise the Erk signalling in response to Fgf8 protein, respectively, in *ex vivo* cultures of living tissue. Examination of endogenous Fgf8 protein and Erk signalling outputs in *Hs2st^−/−^* and *Hs6st1^−/−^* embryos suggests that our *ex vivo* findings have physiological relevance *in vivo*. Our discovery identifies a new class of mechanism to tune Fgf8 function by regulated expression of Hs2st and Hs6st1 that is likely to have broader application to the >200 other signalling proteins that interact with HS and their function in neural development and disease.

## Abstract (precis)

**Summary:** Regulating the fine structure of cell surface carbohydrates provides a mechanism for fine tuning the formation of morphogen gradients in the embryonic mouse fore-brain.

## INTRODUCTION

Fibroblast growth factors (FGFs) are secreted signalling proteins that function as morphogens by forming protein concentration gradients emanating from focal sources to elicit dose-dependent outcomes (Itoh and Ornitz, 2004; Guillemot and Zimmer, 2011). The broad functional importance and evolutionarily antiquity of FGFs in neural development implies that molecular mechanisms regulating FGF morphogen gradients must be both robust and flexible – robust to reproducibly generate brains during embryogenesis, yet flexible to allow context-specific function as well changes to neural molecular biology over evolutionary timescales. To these ends, could context-specific FGF regulators act on relatively invariant core system FGF signalling components, FGF proteins, and their receptors?

The classic ‘source-sink’ model uses differential equations to describe how steady-state protein concentration gradients, formed by protein spreading from a focal source through cell fields, require simultaneous diffusion through the tissue, and clearance (the sink) from the tissue. According to the model, reaching a steady-state protein concentration gradient is key for the interpretation of the gradient by cells (Crick, 1970). FGF protein gradients elicit intracellular response when FGFs bind cell surface FGF receptors (FGFRs) to trigger the intracellular extracellular signal regulated kinase/mitogen activated protein kinase (ERK/MAPK) pathway, culminating in activating phosphorylation (ERK→pERK). The negatively charged heparan sulphate (HS) glycosaminoglycan is a component of cell surface and extracellular matrix (ECM) heparan sulphate proteoglycans (HSPGs). HS is well placed to play regulatory roles in FGF gradient formation and signalling by binding FGFs in the ECM and as an obligate FGF co-receptor in ternary FGF:FGFR:HS signalling complexes (Carlsson and Kjellen, 2012; Bülow and Hobert, 2004). HS promiscuously restricts net spreading of FGFs (Fgf2, Fgf4, Fgf8, and Fgf10) by hindering diffusion and modulating the receptor-mediated endocytosis (RME) mediated sink by binding FGFs to the cell surface (Shimokawa et al., 2011; Muller et al., 2013; Schlessinger et al., 2000; Loo and Salmivirta, 2002; Ornitz et al., 1992; Allen and Rapraeger, 2003; Yu et al., 2009; Qu et al., 2012; Duchesne et al., 2012). HS synthesis is a multistep enzymatic process where regulating one or more steps could control FGF morphogen function. The linear [uronic acid – glucosamine]_n_ HS polymer is synthesised by EXT enzymes and modified by enzymatic addition and removal of sulphate groups by heparan sulphotransferases (HSTs) and sulphatases (SULFs), respectively. HS modifying enzymes underwent evolutionary expansion from the origin of multicellular animals suggesting a role in evolving tissue complexity. Mammals have 17 HS modifying enzymes classed into five categories (HS3ST, NDST, HS2ST, HS6ST, and SULF) according to exactly where on the uronic acid–glucosamine disaccharide residue they add or remove sulphate groups. Many HS modifying enzymes are expressed in developing brain, pointing to functional importance in neural development (Carlsson and Kjellen, 2012; Kreuger and Kjéllen, 2012; Ori et al., 2011). Furthermore, a screen of different HS structures prepared *in vitro* showed that different HS structures selectively activate Fgf signalling in Fgf signalling bioassays in cell lines (Guimond and Turnbull, 1999). Although there have been many studies on the role of HS in modulating the Fgf signalling gradients, the role of different HS modifying enzymes is not really understood especially in the physiological context of living tissue. Regulating FGF signalling by modulating HS synthesis, for example via EXT expression, is likely a blunt strategy because promiscuous interaction of HS with multiple paracrine FGFs and other signalling proteins incurs risk of off-target effects. Could an alternative mechanism harnessing differential HS sulphation generated by differential expression of HS modifying enzymes deliver more nuanced control of FGF function in developing brain? Here we investigate this question for two HS modifying enzymes widely expressed in developing brain that catalyse two distinct forms of HS sulphation – Hs2st adds sulphate to the carbon atom in position 2 of uronic acid (2-*O* HS sulphation), while Hs6st1 sulphates position 6 of glucosamine (6-*O* HS sulphation). We turned to the tractable system provided by Fgf8 signalling in the ventricular zone (VZ) of the cortico-septal boundary (CSB) at the developing mouse embryonic day (E)14.5 telencephalic midline to investigate the function of *Hs2st* and *Hs6st1* in Fgf8 gradient formation, and the Erk response to Fgf8 in living tissue. Severe developmental phenotypes map to the CSB when FGF8/Fgf8 or HS are disrupted in humans or mice, indicating their functional importance in this region (Inatani et al., 2003; Smith et al., 2006; Suzuki-Hirano and Shimogori, 2009; Chan et al., 2015; Mason, 2007; Tornberg et al., 2011; Johnson et al., 2004). *Hs2st^−/−^* and *Hs6st1^−/−^* embryos exhibit hyperactive Erk signalling triggering precocious glial guidepost cell migration from the VZ to ectopic midline positions where they subsequently misdirect corpus callosal axons, a phenotype linked to elevated Fgf8 levels in *Hs6st1^−/−^* but not *Hs2st^−/−^* embryos (Clegg et al., 2014; Conway et al., 2011). This work prompts the hypothesis that *Hs2st* and *Hs6st1* differentially regulate Fgf8 function. Here we employ quantitative *ex vivo* analysis and, consistent with this hypothesis, show that Hs2st and Hs6st1 each exert significantly different control over the emergence of a steady-state Fgf8 protein concentration gradient and the intracellular pErk response to Fgf8 in living tissue.

## RESULTS

### *Ex vivo* assay for Fgf8 gradient formation

Fgf8 gradient formation involves the production of Fgf8 at the source, followed by Fgf8 transport through the extracellular matrix in the tissue, activation of the signalling pathway through receptor binding of responding cells, and finally Fgf8 protein clearance. The emergence of the Fgf8 protein gradient could be modulated by HS modifying enzymes at any step of the pathway. Dissecting the role of *Hs2st* and *Hs6st1* in Fgf8 gradient formation modulation *in vivo* is difficult as there are many variables that are not tenable to experimental control, such as the amount of Fgf8 protein emanating from the source. Therefore, we designed an *ex vivo* bead assay in which we challenged slices of embryonic forebrain in which HS was enzymatically removed (Heparanase treatment) or lacking functional HS modifying enzymes Hs2st (*Hs2st*^−/−^ tissue) or Hs6st1 (*Hs6st1^−/−^* tissue) with a constant amount of Fgf8 to probe the formation of a Fgf8 protein gradient through time. The *ex vivo* assay allowed us to probe Fgf8 gradient emergence in much smaller timescales, that is impossible to assay *in vivo*, providing a higher temporal resolution of Fgf8 gradient formation.

**Fig. 1.**
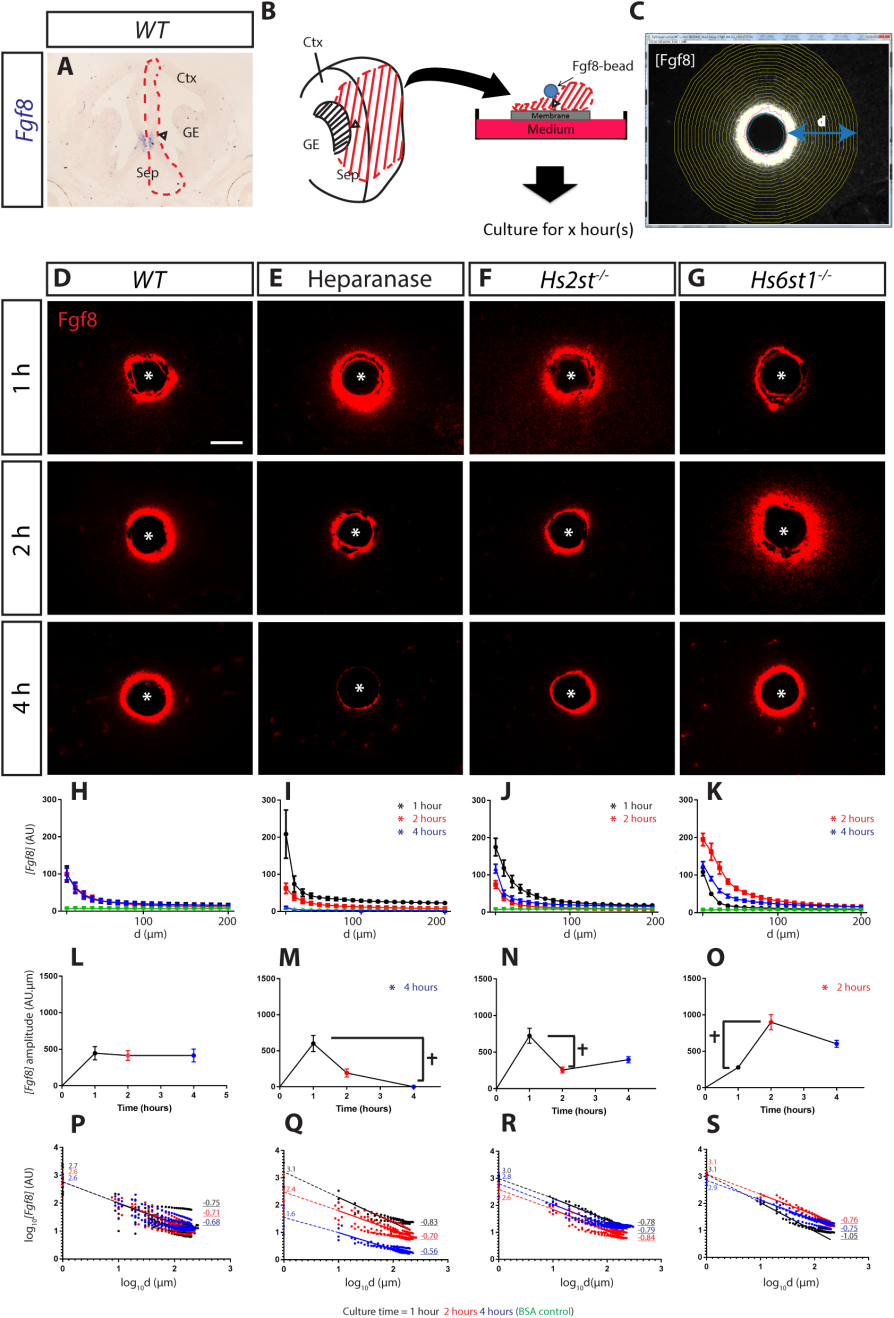
**Differential HS sulphation regulates *[Fgf8]* gradient formation with differential kinetics.** (A) *Fgf8* mRNA is located at the CSB angle (arrowhead). This image is also shown enlarged in Fig. 3C. Sep, septum; Ctx, cortex; GE, ganglionic eminences. (B) Schematic of *ex vivo* explant culture with Fgf8-infused bead implanted at the CSB angle (arrowhead). (C) Fgf8 immunofluorescence (here shown as greyscale) illustrating concentric rings (yellow) used to quantify *[Fgf8]* at increasing distance (d) from the Fgf8-bead edge. (D-G) Representative images of Fgf8 immunofluorescence (red) in sections though cultured explants under different conditions and time-points indicated next to panels, asterisk marks bead centre. (H-K) *[Fgf8]* gradient up to 200 µm from the bead. Data for 1, 2, and 4 h time-points coloured black, red, and blue respectively with BSA control data coloured green, asterisks indicate significant difference between each particular condition with its corresponding *WT* (Kolmogorov–Smirnov test; *P*<0.05 following a Bonferroni correction for multiple pairwise comparisons). (L-O) Total Fgf8 level within 200 µm of the bead. Cross indicates significant differences between bracketed time-points within a culture condition indicating fluctuating amplitude through time, while asterisks indicate significant difference at a particular time-point between each particular condition and *WT* (two-way ANOVA followed by Holm-Sidak post hoc test; *P*<0.05). (P-S) Fitted curve of H-I in *log_10_[Fgf8] vs. log_10_[d]*. Each plot shows all data points and average line of best fit (solid line) with dotted line indicating extrapolation to *log­_10_[Fgf8]* axis with numbers on indicating intercept and underlined numbers the slope. Number of explants analysed (N): *WT*, 8; Heparanase, 5; *Hs2st^−/−^*, 5; *Hs6st1^−/−^*, 4. Scale bar in D applies to D-G: 100 µm. In H-S values are shown as mean±s.e.m.

In E14.5 mouse telencephalon, *Fgf8* mRNA expression is restricted to the angle between the cerebral cortex and the septum at the CSB, identifying this region as the source of Fgf8 protein *in vivo* (Fig. 1A, arrowhead marks angle). We recreated this configuration *ex vivo* by embedding beads infused with recombinant Fgf8 protein (or BSA control) into the VZ at the CSB angle of telencephalic midline tissue explants (dissected region indicated by red dashed line in Fig. 1A and red shading in schematic in Fig. 1B, arrowheads marks angle). Explants were cultured for 1-4 h to allow Fgf8 protein to spread from the bead into the surrounding living tissue, after which explants were fixed and sectioned, reacted for Fgf8 immunofluorescence, imaged, and IMAGEJ used to quantify how Fgf8 protein concentration (*[Fgf8]*) varies with distance (*d*) from the Fgf8-bead edge (Fig. 1C; Fig. S1).

### Hs2st and Hs6st1 differentially regulates Fgf8 gradient formation *ex vivo*

When wild-type (*WT*) tissue was challenged with an Fgf8-soaked bead, the concentration of Fgf8 protein (*[Fgf8]*) is highest closest to the Fgf8-bead and decays with increasing distance from the bead edge (Fig. 1D). Plotting *[Fgf8]* [expressed as arbitrary units (AU)] in the tissue against distance from the bead at each time-point produces classic decay curves with *[Fgf8]* falling non-linearly with increasing distance from the bead. Comparison of these curves shows no change over the 1-4 h period, indicating that a steady state gradient is achieved <1 h after introducing the Fgf8-bead into the tissue [Fig. 1H – note superimposition of black (1 h), red (2 h), and blue (4 h) lines]. A Kolmogorov–Smirnov (KS) test was used to compare *WT* spatial concentration curves over 1-4 h revealing no statistically significant differences for any *WT*-*WT* comparison, and confirming that the gradient reaches a steady-state before 1 h (Table S1A). Note these experiments only measure Fgf8 exogenously supplied by the bead; we did not detect any endogenous Fgf8 in the slices as no Fgf8 signal was detected in the BSA bead controls (green lines in Fig. 1H-K). We suspect that the endogenous Fgf8 in the slices were cleared during slice preparation when slices were incubated in recovery medium for >1 h prior to bead implantation. Next we assessed the role of HS and the activity of Hs2st and Hs6st1 on the *[Fgf8]* gradient over time by removing HS (pre-treating *WT* tissue with heparanase) or using tissue from *Hs2st^−/−^* or *Hs6st1^−/−^* embryos. Representative images of Fgf8 immunofluorescence show that these three conditions resulted in an unstable *[Fgf8]* gradient that does not achieve the WT steady state between 1 and 4 h (compare *[Fgf8]* between time-points in Fig. 1E-G). The *[Fgf8]* gradient of each condition also deviated from *WT [Fgf8]* (compare *[Fgf8]* in Fig. 1D to those in E-G at each time-point).

In heparanase-treated, *Hs2st^−/−^*, and *Hs6st1^−/−^* experiments, in contrast to *WT*, there were multiple significant differences between the gradients at successive time-points (shown as t, Fig. 1M-O; KS test *P*-values for within condition comparisons between spatial concentration curves listed in Table S1A) indicating impaired ability for forming a steady state *[Fgf8]* gradient. There were also significant deviations from the *WT [Fgf8]* gradient, although here, heparanase-treated, *Hs2st^−/−^*, and *Hs6st1^−/−^* experiments showed different effects. Following heparanase treatment, the spatial concentration curve was higher than *WT* after 1 h and then dropped rapidly, becoming lower than *WT* by 2 h and barely detectable after 4 h, all of these differences were significant. Both *Hs2st^−/−^* and *Hs6st1^−/−^* tissue also formed non-steady-state *[Fgf8]* gradients. However it is important to note that the *[Fgf8]* gradient in *Hs2st^−/−^* tissue was not significantly different to the *WT [Fgf8]* gradient after 4 h in culture, although the *[Fgf8]* gradient fluctuated in the earlier time-points. The spatial concentration curve formed in *Hs2st^−/−^* tissue was significantly higher than *WT* after 1 h, lower than *WT* after 2 h, and not significantly different to *WT* after 4 h (Fig. 1J). On the other hand, the *[Fgf8]* gradient in *Hs6st1^−/−^* tissue remained higher than the *WT* gradient from the second time-point and throughout the culture. The spatial concentration curve formed in *Hs6st1^−/−^* tissue was not significantly higher than *WT* after 1 h, but then became much higher at 2 h and then fell, but remained significantly higher than *WT* by 4 h (Fig. 1K).

These data demonstrate that the formation of a steady-state *[Fgf8]* gradient over a 4 h time period is sensitive to the presence of HS (heparanase experiment) (Fig. 1E,I,M,Q), and to Hs2st and Hs6st1 activity (*Hs2st^−/−^* and *Hs6st1^−/−^* experiments) (Fig. 1F,G,J,K,N,O,R,S).

### Hs2st and Hs6st1 regulates formation of a stable Fgf8 gradient via modulation of *[Fgf8]* amplitude but not *[Fgf8]* decay

The *[Fgf8]* gradient is determined by three independent factors: *[Fgf8]* at the source; the amount of Fgf8 protein in the gradient (the amplitude); and the rate at which *[Fgf8]* decays with increasing distance from the source (the decay). In these experiments *[Fgf8]* at the source is constant (see below). We next asked whether the HS manipulations contribute to changing the *[Fgf8]* gradient by modulating its amplitude, decay, or both.

We calculated the amplitude of the *[Fgf8]* gradient from the area under the curves in Fig. 1H-K and presented the amplitude variation with time in Fig. 1L-O. Statistical comparisons were made to assess whether amplitude achieved steady state and deviated from *WT* (two-way ANOVA followed by post hoc Holm-Sidak test; *P*-values for these pair-wise comparisons listed in Table S1B). Comparing *[Fgf8]* amplitude between time-points of each condition, the amplitude of *WT* tissue remained constant over time with no significant differences, indicating a steady-state amplitude was achieved as early as 1 h of culture (Fig. 1L). In contrast, there are significant differences between some time-points in heparanase-treated, *Hs2st^−/−^*, and *Hs6st1^−/−^* experiments indicating an impaired ability to maintain a steady-state Fgf8 amplitude over time (Fig. 1M-O, cross marks significant differences between time-points). There were also significant differences in amplitude when comparing HS manipulated cultures to *WT* cultures (Fig. 1M-O, asterisk marks time-points where the amplitude is significantly different to *WT*). Heparanase treatment resulted in amplitude not significantly different to *WT* for the first 2 h followed by a significant drop to very close to detection threshold (Fig. 1M). We found that the amplitude fluctuated significantly in *Hs2st^−/−^* tissue; however, none of these changes were significantly different to corresponding *WT* time-points (Fig. 1N). Nevertheless, the significant difference between the *Hs2st^−/−^* and *WT [Fgf8]* curves at both 1 and 2 h time-points suggests that failure to attain a stable *[Fgf8]* amplitude is causing significant changes to the *[Fgf8]* gradient. In contrast, *Hs6st1^−/−^* tissue showed a significant 2.5-fold increase in amplitude after 2 h, which then fell by 4 h when it was not significantly different to *WT* (Fig. 1O).

The decay of a protein gradient is described by different equations depending on both diffusion kinetics and whether the degradation rate, for example clearance of Fgf8 by RME, is linear or non-linear as a function of distance from the source (Wartlick et al., 2009; also see Discussion). We found that the *[Fgf8]* spatial distribution in our experiments behaved as power law gradients, with *log[Fgf8] vs log distance* transforming the *[Fgf8] vs distance* curves to linearity (R^2^>0.9, Table S1C); the more negative the slope the more rapid the *[Fgf8]* decay as a function of distance (Fig. 1P-S, underlined numbers to right of each line indicate average slope values). Statistical comparison of the slopes showed that there were no significant differences between *WT* and any HS condition at any time-point (Table S1C), indicating that none of the HS manipulations described here have a significant impact on *[Fgf8]* decay as a function of distance from the source. Interestingly, although the gradient of *Hs6st1^−/−^* was significantly different from *WT* at the 4 h time-point, neither the *[Fgf8]* amplitude or *[Fgf8]* decay (slope in Fig. 1S) of the 4 h time-point was significantly different from *WT*. In addition, we also observed this phenomenon with the *Hs2st^−/−^ [Fgf8]* gradient curves at the 1 and 2 h time-points. Presumably, both are contributing to affect the Fgf8 gradient with neither contribution reaching statistical significance. We also noted that although the *Hs6st1^−/−^* slopes were found to be not significantly different between different time-points, there is a trend that the *Hs6st1^−/−^* slopes between time-points were not parallel (Fig. 1S). This was only observed in the *Hs6st1^−/−^* slices as *Hs2st^−/−^* and *WT* slices have parallel slopes between different time-points (compare Fig. 1S with Fig. 1P-R), while slopes for heparanase treated slices were not parallel due to the lack of an Fgf8 gradient after 4 h in culture. The *log[Fgf8]* intercept, obtained by extrapolation (dotted lines in Fig. 1P-S, numbers on the *log[Fgf8]* axis indicate average intercept values), gives *[Fgf8]* at the source which is the surface of the Fgf8 bead (<1 µm from bead edge), and there were no significant differences (two-way ANOVA flowed by post hoc Holm-Sidak testing of pairwise comparisons, Table S1D) except between *WT* and heparanase treated tissue at the 4 h time-point. This indicates the bead *[Fgf8]* was constant in all conditions and time-points (except heparanase treated cultures after 4 h). This was confirmed by direct quantification of bead *[Fgf8]* (Fig. S1E), so bead *[Fgf8]* was not a variable contributing to differences observed between other conditions and time-points.

To conclude, the rapid (<1 h) formation of a steady state *[Fgf8]* gradient in *WT* tissue is dependent on the presence of HS and the activity of HS modifying enzymes Hs2st and Hs6st1. The formation of *[Fgf8]* steady-state gradient can be primarily attributed to a role for HS in controlling the amount Fgf8 protein present in the concentration gradient (*[Fgf8]* amplitude), as opposed to modulating the decay of Fgf8 as a function of distance from the source (*[Fgf8]* decay). Critically, our analysis found that Fgf8 behaved differently in living *Hs2st^−/−^* and *Hs6st1^−/−^* tissue with a much stronger effect in *Hs6st1^−/−^* tissue, indicating that the Hs6st1 plays the dominant role in supressing Fgf8 levels.

### Hs2st and Hs6st1 differentially regulates pErk response to the Fgf8 protein gradient *ex vivo*

**Fig. 2.**
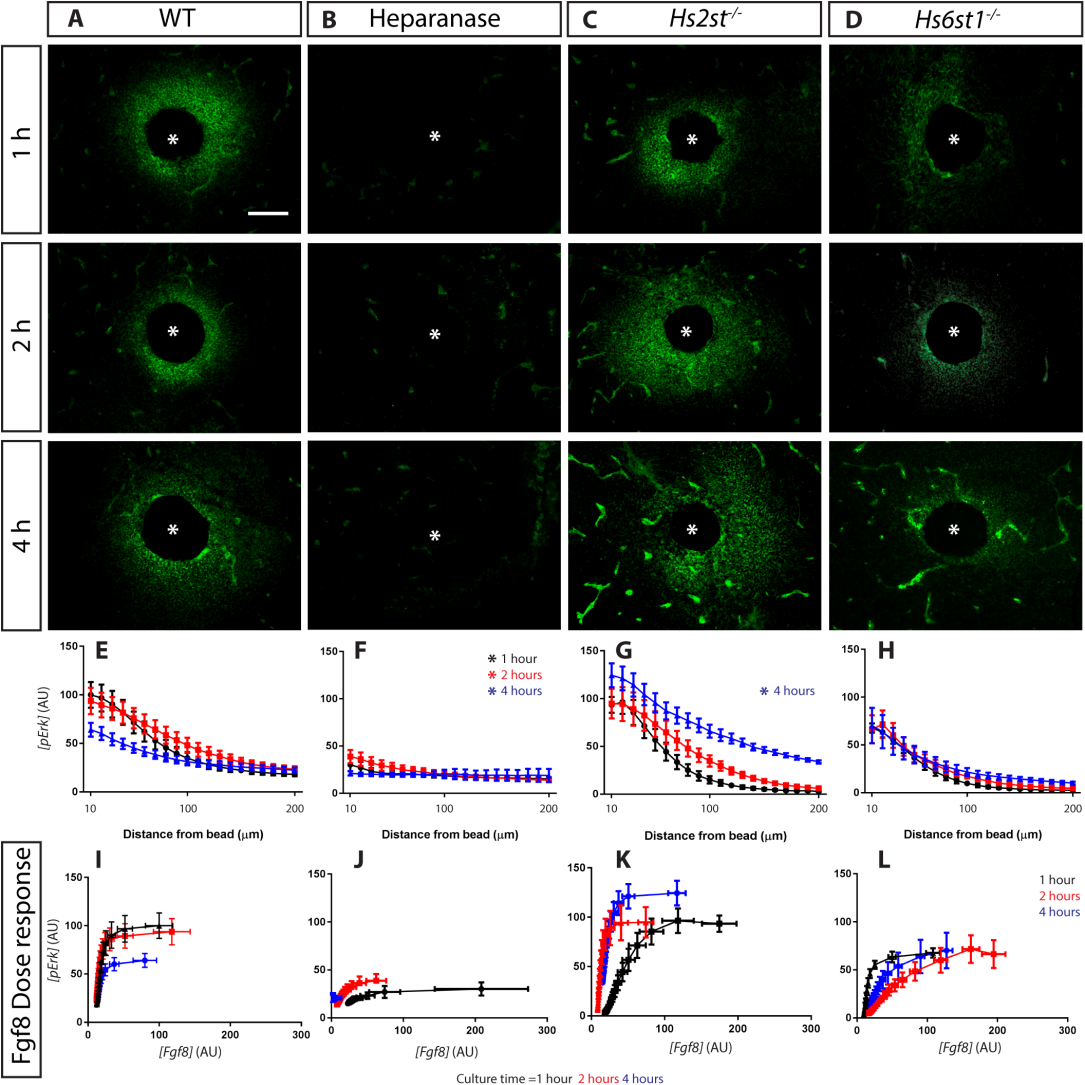
**Differential HS sulphation differentially regulate Erk response to Fgf8.** (A-D) Representative images of pErk immunofluorescence in sections though cultured explants representing different conditions and time-points indicated on panels, asterisk marks bead centre. (E-H) *[pErk]* gradient formed at different time points up to 200 µm from the bead. Asterisks indicate significant difference between each particular condition with its corresponding *WT* [Kolmogorov–Smirnov test (*P*<0.05) following a Bonferroni correction for multiple pairwise comparisons]. (I-L) *[Fgf8]/[pErk]* dose response curves. Number of explants analysed: *WT*, 8; Heparanase, 5; *Hs2st^−/−^*, 5; *Hs6st1^−/−^*, 4. Data for 1, 2, and 4 h time-points coloured black, red, and blue respectively. Scale bar in D applies to A-D: 100 µm. In E-L values are shown as mean±s.e.m.

The binding of Fgf8 to its receptor activates the phosphorylation of Erk. As HS also functions as an Fgf co-receptor, we next investigated how HS, Hs2st and Hs6st1 regulated the Erk response to the Fgf8 gradient (Fig. 1B). All sections reacted for Fgf8 immunofluorescence (Fig. 1, red) were simultaneously reacted for pErk immunofluorescence (Fig. 2, green). In *WT* cultures, pErk forms a gradient with the highest concentration of pErk (*[pErk]*) closest to the bead (Fig. 2A show representative images with gradient quantification in Fig. 2E). There is a marked drop in the *[pErk]* gradient after 4 h (red=black>>blue lines in Fig. 2E; Table S2). Our analysis quantifies both Fgf8 and pErk signals at each distance from the Fgf8-bead, and these values were combined in a *[pErk] (y-axis) vs [Fgf8] (x-axis)* plot to generate a dose response curve showing the pErk response elicited by different *[Fgf8]* in the tissue (Fig. 2I). The *[Fgf8]/[pErk]* dose response is stable for the first 2 h, but after 4 h the cells becomes much less sensitive with saturating levels of Fgf8 eliciting ∼60% the pErk output of the earlier time-points (blue<red=black lines in Fig. 2I).

Eliminating HS altogether (heparanase treatment) significantly reduces the *[pErk]* gradient compared to *WT* at all time-points (Fig. 2B shows representative images with pErk distribution curves in Fig. 2F, asterisk marks two-way ANOVA followed by post hoc Holm-Sidak test *P*<0.05), and the *[Fgf8]/[pErk]* dose response shows strong de-sensitisation to Fgf8 (Fig. 2J). In contrast, loss of Hs2st or Hs6st1 in *Hs2st^−/−^* or *Hs6st1^−/−^* tissue, respectively, has a weaker effect on the *[pErk]* distribution curve, but tellingly each had a distinct effect on the *[Fgf8]/[pErk]* dose response. In *Hs2st^−/−^* cultures, a substantial pErk response surrounding the bead is detectable at all time points (Fig. 2C) and the *[pErk]* gradient increases with time in culture and differs significantly from *WT* after 4 h (black<red<<blue line in Fig. 2G, asterisk marks two-way ANOVA followed by post hoc Holm-Sidak test *P*<0.05). The *[Fgf8]/[pErk]* dose response shows that *Hs2st^−/−^* tissue becomes progressively more sensitive to Fgf8 over time (black<red<blue line in Fig. 2K). A completely different phenotype occurs in *Hs6st1^−/−^* cultures (Fig. 2D) where the *[pErk]* gradient is similar across all time points (black=red=blue lines in Fig. 2H) and not significantly different to *WT*. The *[Fgf8]/[pErk]* dose response shows that *Hs6st1^−/−^* tissue is de-sensitised to Fgf8 and saturating doses of Fgf8 only elicited ∼60% of the pErk response of *WT* cultures after 1 and 2 h (compare Fig. 2I to L). We conclude that HS facilitates the pErk response to Fgf8 while Hs2st and Hs6st1 have complementary functions by negatively and positively modulating the *[Fgf8]/[pErk]* dose response respectively.

### Hs2st and Hs6st1 differentially regulates Fgf8 and pErk *in vivo*

The *ex vivo* experiments showed us how living tissue reacts when challenged with an implanted bead acting as an experimentally introduced source of Fgf8, likely at non-physiological levels. HS was found to be critical for *[Fgf8]* gradient formation and the Erk response to Fgf8 as the loss of HS resulted in no detectable *[Fgf8]* gradient after 4 h in culture and loss of Erk response to Fgf8. *Hs2st* was found to play a minor role in the maintaining a stable Fgf8 gradient as removal of *Hs2st* resulted in *[Fgf8]* fluctuations during *[Fgf8]* gradient formation before eventually reaching *WT [Fgf8]* levels, albeit taking a longer time than *WT* tissue. Meanwhile, *Hs6st1* was found to be needed to suppress *[Fgf8]* during the *[Fgf8]* gradient formation. The *ex vivo* experiments also showed that *Hs2st* antagonises the Erk response to Fgf8, while *Hs6st1* is needed to promote Erk response to Fgf8.

**Fig. 3.**
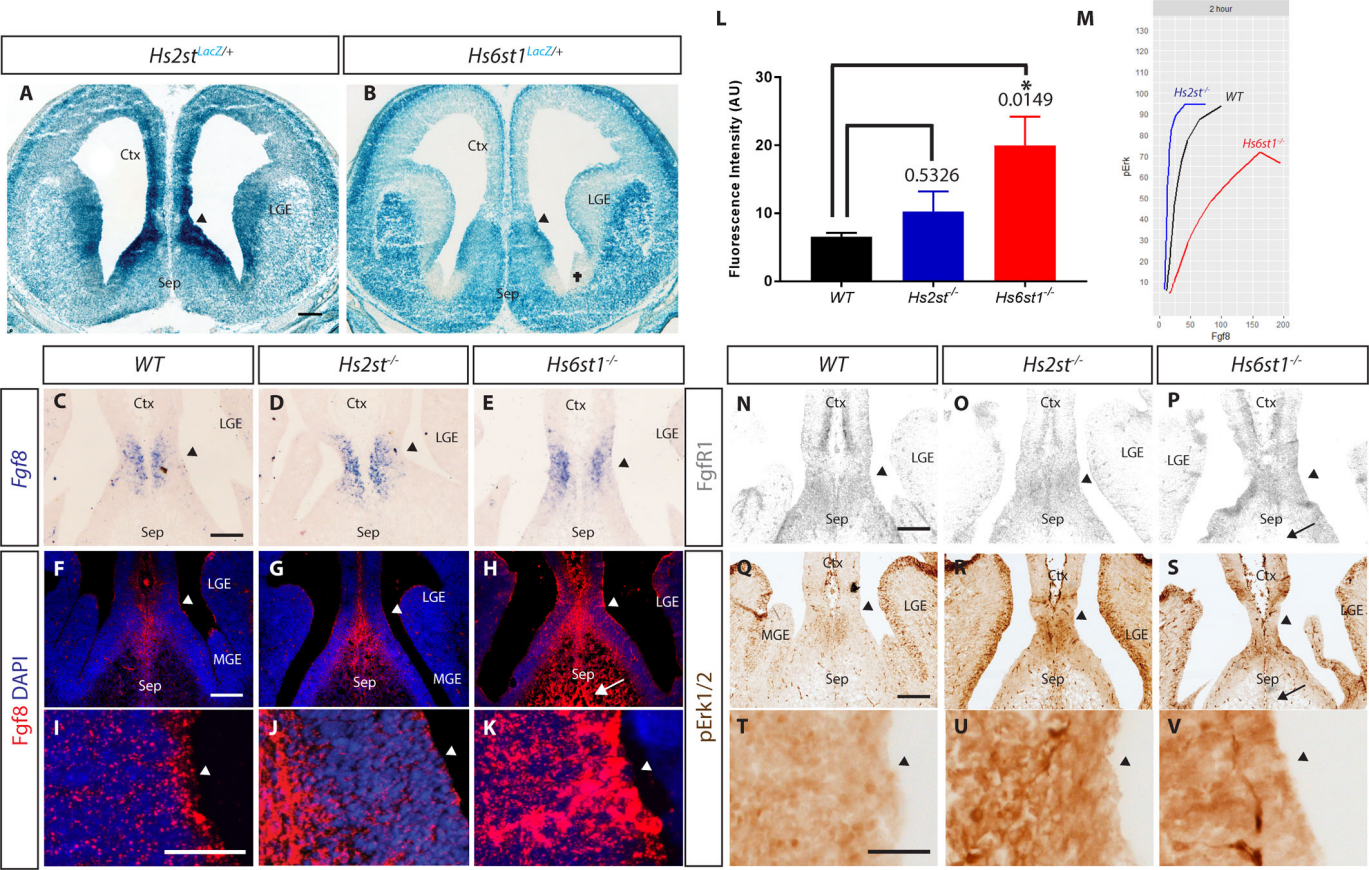
**Analysis of Fgf8/Erk signalling components in the CSB region of E14.5.** *WT*, *Hs2st^−/−^*, and *Hs6st1^−/−^* embryos show correlation to *Hs2st* and *Hs6st1* action *ex vivo*. (A) *Hs2st* and (B) *Hs6st1* expression visualized by LacZ staining of *Hs2st^LacZ/+^* and *Hs6st1^LacZ/+^* sections, respectively. Cross marks an area with very low *Hs6st1^LacZ^* expression, *N*=4. (C-E) *Fgf8* mRNA expression. *N*=5. Note that the image in C is an enlargement of that shown in Fig. 1A. (F-H) Fgf8 protein expression. *N*=3. (I-K) Higher magnification of VZ angle in F-H. (L) Quantification of Fgf8 fluorescent intensities in I-K. *P* values are as depicted on graph. Error bars indicate standard error of mean. (M) Dose response of *WT*, *Hs2st^−/−^*, *Hs6st1^−/−^* tissue showing increased sensitivity to Fgf8 in *Hs2st^−/−^* while *Hs6st1^−/−^* tissue have decreased sensitivity to Fgf8 when compared to *WT* tissue. (N,O) Expression of Fgfr1 protein. *N*=4 for *WT*, *N*=3 for *Hs2st^−/−^* and *Hs6st1^−/−^*. (Q-S) pErk protein expression. *N*=3. (T-V) Higher magnification of ventricular zone in Q-S. In A, B, C-K and N-V arrowheads indicate apical surface of the ventricular zone at the CSB angle; arrows in H, P, S indicate Fgf8^High^, Fgfr1^Low^, pErk^Low^ septal area in *Hs6st1^−/−^* embryos. *N*=3. Ctx, cortex; LGE, lateral ganglionic eminence; MGE, medial ganglionic eminence; Sep, septum. Scale bars: A applies also to B: 200 µm; C applies to C-E: 100 µm; F applies to F-H: 100 µm; I applies to I-K: 100 µm; N applies to N-P: 100 µm; and Q applies to Q-S: 100 µm; T applies to T-V: 100 µm.

Do the principles we have established *ex vivo* for the differential control exerted by Hs2st and Hs6st1 on the Fgf8 protein concentration gradient and the pErk response it elicits apply *in vivo*? In order to address this, we examined the expression of Hs2st, Hs6st1, Fgf8, Fgfr1, and pErk in the CSB region of E14.5 *WT*, *Hs2st^−/−^*, and *Hs6st1^−/−^* embryos. *Hs2st* and *Hs6st1* are expressed at relatively high levels in the VZ at the CSB angle (black arrowheads in Fig. 3A,B). Note that compared to their high expression at the CSB angle, *Hs2st* is expressed at much lower levels in lateral telencephalon (Fig. 3A) and *Hs6st1* is expressed at much lower levels in the area marked with a cross in Fig. 3B. There is no gross difference in *Fgf8* mRNA distribution between *WT*, *Hs2st^−/−^*, or *Hs6st1^−/−^* CSB (Fig. 3C-E), indicating any differences in Fgf8 protein distribution between genotypes stems from post-transcriptional regulation. Fgf8 protein is found at low levels in the *WT* VZ, predominantly at the apical surface, and is more abundant at the midline (Fig. 3F with higher magnification of VZ in I). Fgf8 protein is increased at the *Hs2st^−/−^* midline VZ (Fig. 3G with higher magnification of VZ in J). By far the strongest effect on Fgf8 protein distribution is seen in *Hs6st1^−/−^* CSB where, in addition to an increase at the midline and expansion into the septum (arrow in Fig. 3H), there is much stronger staining throughout the VZ (Fig. 3H with higher magnification of the VZ in K). Quantifying the fluorescence intensity of Fgf8 immunofluorescence of the VZ of the CSB (Fgf8 signal quantified in a 100 µm×180 µm box placed over the CSB encompassing area shown in Fig. 3I-K) enabled us to measure the *[Fgf8]* at the CSB *in vivo*. We found that there was a statistically significant threefold increase of Fgf8 fluorescence in *Hs6st1^−/−^* tissue; however, there was no statistically significant increase in the *Hs2st^−/−^* tissue compared to *WT* (Fig. 3L). These results correlated with the *ex vivo* results previously obtained (Fig. 1), in which *Hs6st1* plays a more significant role on *[Fgf8]* gradient amplitude regulation than *Hs2st* at the VZ of the CSB. How do the changes in Fgf8 protein in *Hs2st^−/−^* and *Hs6st1^−/−^* embryos impact on the pErk response? The pErk levels are higher in both *Hs2st^−/−^* and *Hs6st1^−/−^*mutants than in the *WT* (Fig. 3Q-S with higher magnification of the VZ shown in T-V). The intensity of pErk (*Hs2st^−/−^*=*Hs6st1^−/−^* >*WT*, Fig. 3Q-S) staining does not reflect the levels of Fgf8 protein (*Hs6st1^−/−^* >*Hs2st^−/−^* WT, Fig. 3F-L) indicating that WT, *Hs2st^−/−^*, and *Hs6st1^−/−^* tissue is differentially sensitive to Fgf8. These observations can be reconciled if *Hs2st^−/−^* tissue is more sensitive to Fgf8 and *Hs6st1^−/−^* tissue is less sensitive to Fgf8 to that of *WT* tissue, respectively, as we observed in our *ex vivo* experiments (Fig. 3M, also see Discussion). The similar expression of Fgfr1, the principal Fgf8 receptor in this region, between the three genotypes (Fig. 3N-P), indicates differential Fgf8 sensitivity in the VZ is not caused by differential Fgfr1 expression. The lack of Fgfr1 in the septum (arrow in Fig. 3P) likely explains why Erk is not activated in the *Hs6st1^−/−^* septum (arrow in Fig. 3S) despite ectopic Fgf8 in this area (arrow in Fig. 3H).

## DISCUSSION

A general property of HS is reported in other systems retarding the net spread of FGFs through tissue (Shimokawa et al., 2011; Schlessinger et al., 2000; Ornitz et al., 1992; Allen and Rapraeger, 2003; Yu et al., 2009; Qu et al., 2012; Loo and Salmivirta, 2002; Muller et al., 2013; Duchesne et al., 2012). Our heparanase experiments indicate this is likely the case in our system because removing HS causes an initial surge of Fgf8 protein from the Fgf8-bead into surrounding tissue, followed by depletion of Fgf8 from the bead and declining Fgf8 concentration. The main conceptual advance of this study is identifying specific roles for different HS modifying enzymes in *[Fgf8]* gradient formation and function as in *WT* tissues, the time taken to achieve a steady-state gradient is less than 1 h while it took about 4 h to achieve *WT [Fgf8]* gradient in *Hs2st^−/−^* and more than 4 h for *Hs6st1^−/−^* tissues, respectively.

The formation of a steady state protein concentration gradient requires the balancing of three factors: concentration at the source; diffusion through the tissue; and degradation in the tissue. In our *ex vivo* experiments, the source (the Fgf8-bead) provided a constant *[Fgf8]*, so the effects we describe can be attributed to perturbations on Fgf8 diffusion and/or degradation. *[Fgf8]* varies with distance from the source as a power law gradient, a model describing the distribution of a protein diffusing from a source and being cleared from the tissue in a concentration-dependent manner, for example higher Fgf8 concentrations causing increased Fgf8 degradation through an Fgf8-sensing feedback mechanism (Wartlick et al., 2009). We find HS and its sulphation by Hs2st and Hs6st1 are essential for the normal formation and maintenance of a steady-state *[Fgf8]* gradient, because removing HS altogether (heparanase experiment) or leaving HS intact but losing the function of different HS modifying enzymes (*Hs2st^−/−^* and *Hs6st1^−/−^* experiments) all caused significant fluctuations in the gradient amplitude (amount of Fgf8 in the gradient) through time that were not observed in *WT* cultures. Although the fluctuations in Fgf8 gradient amplitude observed in both *Hs2st^−/−^* and *Hs6st1^−/−^* tissue were significant, we observed an initial surge in amplitude at the 1 h time-point in *Hs2st^−/−^*, while the surge was only observed in the 2 h time-point in *Hs6st1^−/−^*. The Fgf8 amplitude of both mutant tissues reached *WT* levels after 4 h in culture. This prompted the question of whether the differences we observed in the Fgf8 gradient formation and the Erk response to the Fgf8 gradient due to the loss of *Hs2st* and *Hs6st1* was due to a timing issue of when steady state is achieved, or that Hs2st and Hs6st1 have fundamentally different roles in the process of establishing the Fgf8 gradient and the Erk response. Analyses of the Fgf8 bead cultures for a longer time period would give further insight into this question, however culturing explants with Fgf8 beads for periods longer than our current study is not viable as Fgf molecules have a relatively short half-life. For example, Fgf10 (which is structurally similar to Fgf8) has a half-life of ∼5 h. This is supported by other studies which performed similar Fgf bead assays using similar time-frames as this study (Harada et al., 2009; Qu et al., 2012). Therefore, bead depletion due to the short half-life of Fgf8 would be a factor affecting the interpretation of results. Furthermore, as discussed above, we have shown that Fgf8 bead depletes to ∼50% in explants that lacks HS in 4 h of culture showcasing the requirement for HS in Fgf8 stability and the short half-life of Fgf8 (Fig. S1E). In contrast to amplitude, the *[Fgf8]* decay, revealed by the slope of *log [Fgf8] vs log distance* power function plots, was not significantly altered by removing HS or losing the function of Hs2st or Hs6st1. This indicates that the mechanism regulating the decay of Fgf8 as a function of distance from the source, including feedback mechanisms regulating decay, were not significantly affected by loss of Hs2st or Hs6st1 function. Net *[Fgf8]* decay is determined both by rates of diffusion and degradation and dissecting the regulation of each component, particularly by Hs6st1 which has the greater effect on Fgf8 concentration, is a topic for future research.

Interestingly, we found that *WT* tissues form a steady-state *[Fgf8]* gradient in <1 h when challenged with an exogenous Fgf8 bead. While the loss of Hs2st affected *[Fgf8]* amplitude stability (Fig. 1N) and loss of Hs6st1 caused increased *[Fgf8]* amplitude (Fig. 1O), we noticed that both *Hs2st^−/−^* and *Hs6st1^−/−^* tissues challenged with an Fgf8 bead eventually approached *WT* levels of *[Fgf8]* amplitude (compare Fig. 1N,O to L 4-h time points). This suggests that for a given dose of Fgf8 input, the CSB tissue tissues must have an inherent *[Fgf8]* gradient it tries to achieve, and Hs2st and Hs6st1 each play different roles in the kinetics of achieving this. This also highlights the damaging loss of control that comes with the loss of Hs2st or Hs6st1 in modulating the timely formation of the Fgf8 gradient, which is critical in a developmental context as information from a morphogen gradient needs to be delivered in the correct developmental time frame. In line with its FGF co-receptor function, we find HS is a pre-requisite for pErk response to Fgf8 (Loo and Salmivirta, 2002; Schlessinger et al., 2000; Pellegrini et al., 2000). Hs2st and Hs6st1 each have more subtle and complementary roles in the *[Fgf8]/[pErk]* dose response. Presumably Hs6st1 and Hs2st are able to function as Fgf8/Erk signalling agonists and antagonists, respectively, through opposing impacts of Hs2st and Hs6st1 catalyzed HS sulphation on the Fgf8:Fgfr1:HS signalling complex. Our finding that Hs6st1 facilitates Fgf8 signalling while repressing Fgf8 levels suggests the hypothesis that Hs6st1-mediated affinity of Fgf8 for the cell surface promotes both clearance by RME and signalling activity, possibly also hindering diffusion, through the common mechanism of stabilising Fgf8:Fgfr1:HS complex formation. It is important to note that there was no difference in the Fgfr1 expression between *Hs2st^−/−^* and *Hs6st1^−/−^* with *WT* (Fig. 3N-P), suggesting that the difference in pErk found in *Hs2st^−/−^* and *Hs6st1^−/−^* (Fig. 3Q-S) are driven by the co-receptor function of HS, which in turn is differentially regulated by *Hs2st* and *Hs6st1.*

Our *ex vivo* experiments revealed that *Hs2st^−/−^* CSB has difficulty maintaining a stable *[Fgf8]* gradient and increased Erk sensitivity to Fgf8, while *Hs6st1^−/−^* CSB has significantly increased Fgf8 levels compared to *WT* and decreased Erk sensitivity to Fgf8. These observations are broadly consistent with our *in vivo* observations at E14.5 where Fgf8 protein levels were elevated in *Hs6st1^−/−^* (but not *Hs2st^−/−^*) CSB in addition to higher Erk activation when compared to *WT*. However, this higher Erk activation was not much higher than the Erk activation in *Hs2st^−/−^* tissue. We reasoned that the increase in Erk activation found in *Hs6st1^−/−^* tissue *in vivo* was due to the increased levels of Fgf8 (∼threefold increase) found *in vivo* (Fig. 3L), despite the tissue being less sensitive to Fgf8. This is also consistent to our previous study at E16.5, where increased Fgf8 protein was only detected in *Hs6st1^−/−^* CSB (Clegg et al., 2014).

In the case of Erk sensitivity to Fgf8, the observed *ex vivo* phenotype anticipated the *in vivo Hs2st^−/−^* and *Hs6st1^−/−^* molecular phenotype in E14.5 telencephalic midline in which, despite *Hs2st^−/−^* having a less Fgf8 protein than *Hs6st1^−/−^* VZ, both shared a rather similar elevation in *[pErk]* relative to *WT*. This uncoupling of the correlation between *[Fgf8]* and *[pErk]* is reconciled by our *ex vivo* finding that *Hs2st^−/−^* cells are sensitised to Fgf8 while *Hs6st1^−/−^* cells are desensitised (Fig. 3M). Using the dose response curve, we can estimate the level of Erk activation of the tissue when presented with a known amount of Fgf8. In the case of *Hs6st1^−/−^* tissue, we can clearly see that a fourfold increase of Fgf8 in *Hs6st1^−/−^* when compared to *WT* would result in higher Erk activation compared to *WT*, even though the sensitivity of *Hs6st1^−/−^* cells to Fgf8 are lower compared to *WT* corroborating our observation *in vivo* (Fig. 3M). While it is difficult to exclude the possibility that other FGF and non-FGF proteins signalling through pErk also contribute to the *Hs2st^−/−^* and *Hs6st1^−/−^* phenotypes, our data show that perturbing the Fgf8/Erk axis makes a major contribution to the observed molecular phenotypes.

It is well known that loss of specific HS sulphation causes compensatory effects. It has been previously shown in *Drosophila* that loss of 2-*O* sulphation causes compensatory increases in 6-*O* sulphation and vice versa, contributing to Fgf signalling in the trachea (Kamimura et al., 2006). There was also a report of increase in N-sulphation compensating for loss of 2-*O* sulphation in Chinese hamster ovary cells (Bai and Esko, 1996). We have previously shown that the *Hs2st^−/−^* mice used in this study have no compensatory increase in other sulphation following the loss of 2-O sulphation (Chan et al., 2015), but the sulphation status of *Hs6st1^−/−^* mice is unknown. However, both *ex vivo* (Figs 1 and 2) and *in vivo* (Fig. 3) results clearly show that any compensation at the sulphation level in *Hs6st1^−/−^* was unable to rescue the increased *[Fgf8]* and Erk desensitisation phenotype, providing further support to the role of differential sulphation in modulating *[Fgf8]* gradient and the Erk response to the *[Fgf8]* gradient. How the molecular phenotypes of *Hs6st1^−/−^* we observed in this study relates to the HS sulphation status awaits biochemical analyses. Unfortunately, it is currently extremely challenging to analyse the sulphation status of tissue as small as our region of interest, the CSB. Nevertheless, the effects of the loss of *Hs2st* and *Hs6st1* observed in this study shed light on the gene function of *Hs2st* and *Hs6st1* in modulating Fgf8 gradient and the Erk response to Fgf8 in the developing telencephalon.

Comparing the consequences of tinkering with overall levels of HS (heparanase treatment) as opposed to its modification (*Hs2st^−/−^* and *Hs6st1^−/−^*) supports the hypothesis that regulating specific HS sulphation via expression of HS modifying enzymes provides a more nuanced mechanism to control Fgf8 morphogen function than globally regulating HS synthesis. Removing HS effectively shuts down Fgf8 function while removing Hs2st or Hs6st1 play more modulatory roles. Furthermore, in contrast to the promiscuous interaction of HS with all paracrine FGFs, we find that Hs2st and Hs6st1 each have distinct effects on Fgf8 and may well have similarly specific relationships with other FGFs, and some of the >200 HS interacting signalling molecules comprising the heparanome in mammals (Ori et al., 2011). In any case, it is clear that robustness of Fgf8 morphogen gradient formation is dependent on appropriate expression of heparan sulphotransferases, providing a hitherto undemonstrated mechanism to flexibly regulate Fgf8 signalling post-transcriptionally. Our discovery establishes a general principle that may well apply more broadly to other HS sulphation modifications and HS interacting signalling molecules and so contribute a deeper understanding of the molecular mechanisms underpinning the development and evolution of multicellular structures. These mechanisms introduce heterogeneity into FGF signalling so are likely a particular asset to the development of a structure as extremely complex as the mammalian brain.

## MATERIALS AND METHODS

### Animals

*Hs2st^LacZ^* (Hs2st^−^) and *Hs6st1^LacZ-IRES-hPLAP^* (*Hs6st1*^−^) mutant alleles were maintained on a CBA background (Leighton et al., 2001; Mitchell et al., 2001; Bullock et al., 1998). Genotyping was performed by as described previously (Pratt et al., 2006; Clegg et al., 2014). All mice were bred in-house in line with Home Office UK legislation. The licenses authorising this work were approved by the University of Edinburgh Review Committee and the Home Office. Animal husbandry was in accordance with the UK Animals (Scientific Procedures) Act 1986 regulations.

### Immunochemistry and LacZ staining

Immunohistochemistry (pErk) and immunofluorescence (Fgf8, pErk, Fgfr1) were performed on *ex vivo* and *in vivo* material as previously described (Clegg et al., 2014; Toyoda et al., 2010). Primary antibodies: rabbit anti-phospho-MAPK1/2 (1/200) (D13.14.4E) (Cell Signalling Technology); mouse rabbit monoclonal antibody anti-Fgf receptor 1 (1/200) (D8E4) (Cell Signalling Technology); mouse anti-10E4 (1/200) (370255-1) (Amsbio); anti-Fgf8 mouse monoclonal antibody (1/2500) (MAB323) (R&D Systems). Secondary antibodies and detection: Peroxidase-conjugated Affinipure goat-anti-mouse IgG_1_ (1/1000); goat anti rabbit Alexa Fluor 488 (1/200) (Invitrogen); and Fgf8 staining was detected via TSA Plus Fluorescence Systems (Perkin-Elmer). LacZ staining performed as described previously (Pratt et al., 2006). *N*≥3 for all genotypes.

### Organotypic explant culture

Modification of methods described by López-Bendito et al. (2006) and Qu et al. (2012). Briefly, explants comprising the CSB region were dissected from E14.5 telencephalon and placed ventricular surface up on a floating membrane with a Fgf8-infused (or BSA control) bead implanted into the CSB angle. *WT*, *Hs2st^−/−^*, or *Hs6st1^−/−^* explants, some of which were pre-treated with Heparanase (Sigma) for 2 h at 37°C, were cultured for 1, 2, or 4 h prior to fixation, sectioning (10 µm frozen sections) and processing for Fgf8 and pErk immunofluorescence and DAPI counterstaining.

### Imaging and analysis

Fluorescent sections were imaged using Leica AF6000 epifluorescence microscope connected to a DFC360 camera on constant exposure settings for three channels, DAPI, Fgf8, and pErk, and analysed using IMAGEJ. IMAGEJ was used to draw 10-µm wide regions of interest (ROI) on each image (see Fig. S1 for illustration of ROI placement) and used to quantify signal intensity at increasing distances from the edge of the bead into the tissue (for Fgf8 and pErk gradient quantification) and in the outer edge of the bead (for Fgf8 only). Data were then combined and graphed using GraphPad Prism 6 (GraphPad). Images from all genotypes of each time point experiment were imaged, processed and analysed in parallel to minimise technical variation for comparison between genotypes of the same time point. Values from BSA control cultures were subtracted to remove background fluorescence. Fgf8 fluorescence in *in vivo* sections were quantified using IMAGEJ. Quantification of Fgf8 *in vivo*: mean fluorescence for boxes of 100×180 µm drawn at the CSB were obtained for at least 2 sections per embryo with N being number of embryos analysed. *WT*, *N*=4; *Hs2st^−/−^*, *N*=3; *Hs6st1^−/−^*, *N*=3.

### Statistical analysis

Statistical comparisons were made between different tissue conditions (*WT*, heparanase treated, *Hs2st^−/−^*, and *Hs6st1^−/−^*) and culture durations (1, 2, and 4 h time-points) using Kolmogorov–Smirnov (KS) test with Bonferroni correction to compare *[Fgf8]* and *[pErk]* gradients and ANOVA followed by Holm-Sidak post hoc test to compare *[Fgf8]* amplitude, *[Fgf8]* at the bead and slopes of Fgf8 distribution. The Fgf8 and pErk fluorescence intensity were normalised to the value at the point closest to the bead of the 1 h time point in *WT* tissue, which was designated as 100%. Statistical comparisons were made between *WT*, *Hs2st^−/−^* and *Hs6st1^−/−^* for Fgf8 fluorescence intensity of *in vivo* sections using one-way ANOVA followed by Dunnett's post hoc test.

## Supplementary Material

Supplementary information
