# Supplementary material for: FGF8 morphogen gradients are differentially regulated by heparan sulphotransferases Hs2st and Hs6st1 in the developing brain
Source: Biol Open. 2017 Nov 20;6(12):1933–42. doi: 10.1242/bio.028605 (PMC5769653; doi:10.1242/bio.028605)
Supplement: Supplementary information [file biolopen-6-028605-s1.pdf]

Supplementary Fig 1

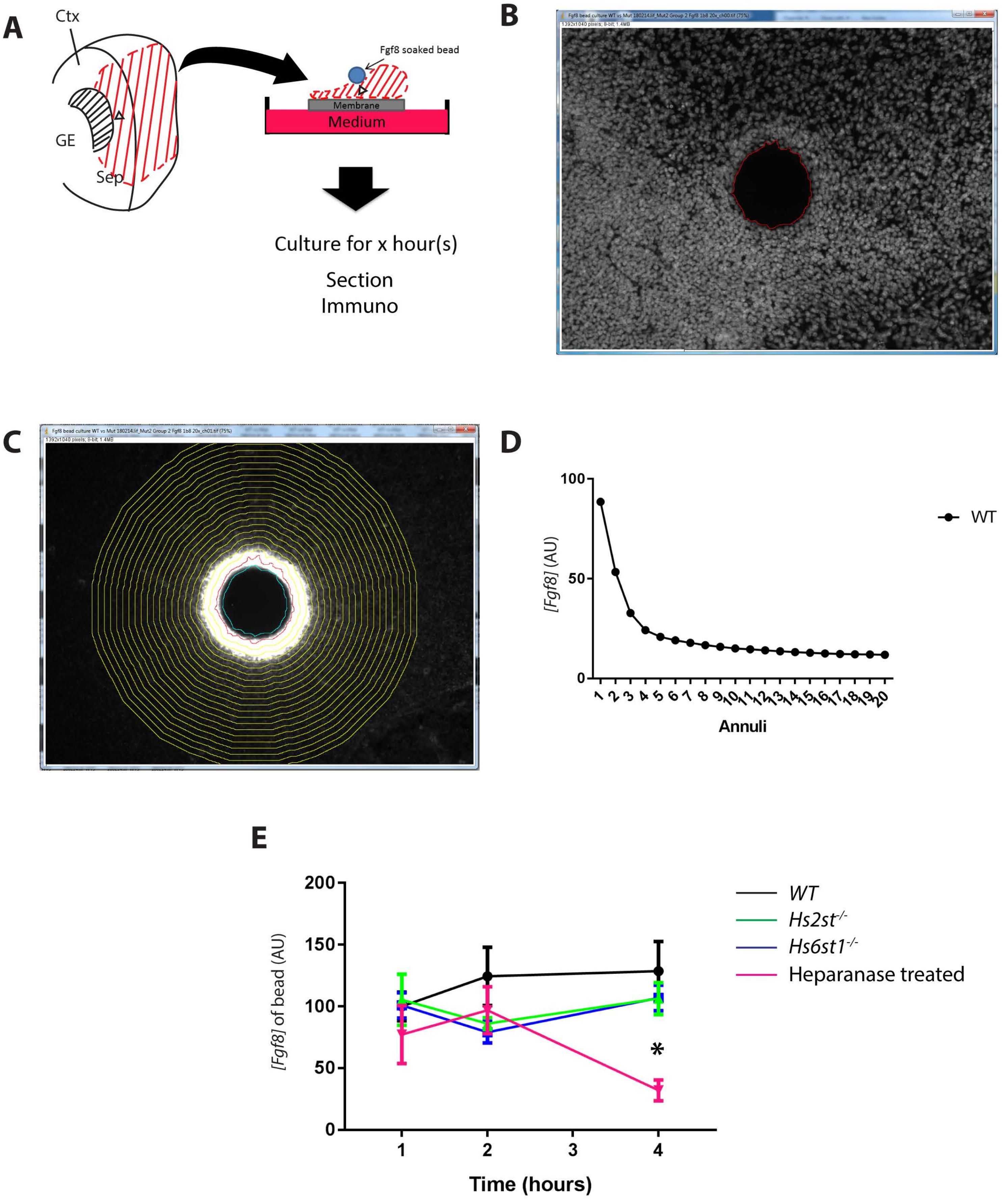

Biology Open • Supplementary information

Supplemental Figure S1

Illustration of gradient quantification method: (A) E14.5 CSB region explant with Fgf8-bead implanted into the angle and cultured before fixation, sectioning, and processing for Fgf8 and pErk immunofluorescence and DAPI. (B, C) DAPI and Fgf8 immunofluorescence channels from a single section. (B) Shows DAPI channel used to identify the interface between the tissue and the bead, marked with a red contour using a freehand tool. (C) In the Fgf8 channel the red contour was extended 10µm inwards (cyan contour) to generate an annulus enclosing the outer rim of the bead infused with Fgf8 protein (for quantifying Fgf8 in the bead) or outwards in successive 10µm steps (yellow contours) to generate a series of nested annuli extending 200µm from the bead edge into the tissue (for quantifying the Fgf8 gradient extending from the edge of the bead into the tissue). (D) Quantification of *[Fgf8]* gradient from section in (C) by plotting average Fgf8 signal in each annulus against distance of the annulus from the edge of the bead. (E) Bead Fgf8 levels analysis in which Fgf8 levels in the bead remains stable from 1 – 4 hours of culture for all genotypes (tested with ANOVA p=0.6126 for wild-type; p=0.2037 for *Hs2st*$^{-/-}$; p=0.5521 for *Hs6st1*$^{-/-}$) except when embedded with tissue treated with heparanase where it decreased to <50% (p=0.0465).

Biology Open • Supplementary information

Supplemental Table 1

(A) KS test results for pairwise comparison of *[Fgf8]* gradients between conditions and time-points. A Bonferoni correction was applied to correct for the 21 informative pairwise comparisons shown in the table, reducing the threshold for statistical significance to p=0.002. (B) Two-way ANOVA with Holm-Sidak posthoc test comparing the *[Fgf8]* amplitude in the tissue of different conditions and time-points. Different conditions were compared to its respective wild-type of the same time-point (highlighted in yellow) and each time-point of a particular condition were also compared against each other (highlighted in aqua). Note that significant p values are in red text and we found a significant effect on the interaction between genotype and time and a significant genotype effect, P<0.0001 and P= 0.0003 respectively. (C) Slope, intercept and r-square values for $log_{10}[Fgf8]$ *vs* $log_{10}\,d$ power law gradient plots. (D) 2-way ANOVA showed no significant variation between slopes P= 0.7754; Condition: P= 0.2927; Time: P= 0.1296 but revealed a significant effect on the interaction between condition and time, a significant effect of condition, and a significant effect of time with P=0.0204, P= 0.003 and P= 0.0036 respectively in $log_{10}[Fgf8]$ intercept. Table shows posthoc Holm Sidak comparisons between *WT* and different HS conditions highlighted in yellow and comparisons between different time-points of a particular condition highlighted in aqua. Note that significant p values are in red text showing the only significant difference is heparanse condition after 4 hours.

Biology Open • Supplementary information

Supplementary Table 1 (a)

Testing for differences in *[Fgf8]* gradient

Condition	WT			Heparanase			*Hs2st*$^{-/-}$			*Hs6st1*$^{-/-}$		
	1	2	4	1	2	4	1	2	4	1	2	4
WT												
1		0.1628	0.2872	<0.0001			<0.0001			0.5358		
2			0.1325		0.0072			<0.0001			<0.0001	
4						<0.0001			0.3446			<0.0001
Heparanase												
1					<0.0001	<0.0001						
2						<0.0001						
4												
Hs2st$^{-/-}$												
1								<0.0001	<0.0001			
2									<0.0001			
4												
Hs6st1$^{-/-}$												
1											<0.0001	<0.0001
2												0.0659
4												

Compare different condition to WT (corresponding time-point)

Comparing time-points (within a condition)

Supplementary Table 1 (b)

Testing for differences in *[Fgf8]* amplitude

Condition	WT			Heparanase			*Hs2st*$^{-/-}$			*Hs6st1*$^{-/-}$		
	1	2	4	1	2	4	1	2	4	1	2	4
WT												
1		0.9999	0.9999	0.982			0.6513			0.9744		
2			0.9999		0.547			0.9505			0.003	
4						0.0379			0.9999			0.6736
Heparanase												
1					0.1092	0.0065						
2						0.9354						
4												
Hs2st$^{-/-}$												
1								0.047	0.4905			
2									0.9744			
4												
Hs6st1$^{-/-}$												
1											0.0025	0.3615
2												0.5908
4												

Compare different condition to WT (corresponding time-point)

Comparing time-points (within a condition)

Supplementary Table 1 (c)

Power law gradient: Slopes, Intercept and R-square values

Condition	Slopes	Intercept $\log_{10}[Fgf8]$	R-square
WT			
1	-0.7499 ± 0.1260	2.698 ± 0.196	0.8854 ± 0.0752
2	-0.7077± 0.0827	2.606 ± 0.1775	0.9445 ± 0.0162
4	-0.6773 ± 0.0852	2.594 ± 0.1791	0.9278 ± 0.0257
Heparanase			
1	-0.8284 ± 0.0989	3.106 ± 0.2675	0.9041 ± 0.0283
2	-0.7017 ± 0.0704	2.366 ± 0.1723	0.9643 ± 0.0108
4	-0.5603 ± 0.1298	1.551 ± 0.2834	0.9356 ± 0.0038
Hs2st$^{-/-}$			
1	-0.7847 ± 0.0098	3.032 ± 0.07831	0.9804 ± 0.0098
2	-0.838 ± 0.0390	2.67 ± 0.09467	0.9401 ± 0.0181
4	-0.7921 ± 0.0575	2.811 ± 0.101	0.9212 ± 0.0215
Hs6st1$^{-/-}$			
1	-1.052 ± 0.0385	3.072 ± 0.0754	0.9735 ± 0.0060
2	-0.7641 ± 0.00741	3.096 ± 0.03592	0.9411 ± 0.0142
4	-0.7478 ± 0.0526	2.865 ± 0.06449	0.9798 ± 0.0038

Biology Open • Supplementary information

Supplementary Table 1 (d)

Testing for differences in *[Fgf8]* at Fgf8 bead (extrapolation of Fgf8 distribution fitted to a power law curve).

Condition	WT			Heparanase			*Hs2st*$^{-/-}$			*Hs6st1*$^{-/-}$		
	1	2	4	1	2	4	1	2	4	1	2	4
WT												
1		>0.9999	>0.9999	0.9991			>0.9999			0.9996		
2			>0.9999		>0.9999			>0.9999			0.9934	
4						0.0198			>0.9999			>0.9999
Heparanase												
1					0.5706	0.0013						
2						0.1801						
4												
Hs2st$^{-/-}$												
1								0.1435	0.3606			
2									0.3606			
4												
Hs6st1$^{-/-}$												
1											>0.9999	>0.9999
2												>0.9999
4												

Compare different condition to WT (corresponding time-point)

Comparing time-points (within a condition)

Supplemental Table 2

KS test results for pairwise comparison *[pErk]* gradients between conditions and time-points. A Bonferoni correction was applied to correct for the 21 informative pairwise comparisons shown in the table, reducing the threshold for statistical significance to P=0.002. Cultures of different conditions compared to its respective *WT* at the same time-point with its p value reported highlighted in yellow while p values reported highlighted in aqua represented cultures within a condition compared to its various time-points. Significant p values are in red text.

Supplementary Table 2

Testing for differences in *[pErk]* gradient

Condition	WT			Heparanase			*Hs2st*$^{-/-}$			*Hs6st1*$^{-/-}$		
	1	2	4	1	2	4	1	2	4	1	2	4
WT												
1		<0.0001	<0.0001	<0.0001			0.3402			0.3695		
2			<0.0001		<0.0001			0.1111			0.8170	
4						<0.0001			0.0008			0.0856
Heparanase												
1					0.0188	0.3256						
2						0.4395						
4												
Hs2st$^{-/-}$												
1								0.0033	<0.0001			
2									0.0611			
4												
Hs6st1$^{-/-}$												
1											0.0049	<0.0001
2												0.0049
4												

Compare different condition to WT (corresponding time-point)

Comparing time-points (within a condition)

Biology Open • Supplementary information
